# P-1293. Molecular and Phenotypic Characterization of ESBL-producing Enterobacter cloacae Complex Isolates Detected at a Single Japanese Hospital Over Seven Years

**DOI:** 10.1093/ofid/ofaf695.1481

**Published:** 2026-01-11

**Authors:** Tetsuya yagi, Keisuke Oka

**Affiliations:** Nagoya University Hospital, Nagoya, Japan., nagoya city, Aichi, Japan; Nagoya University Hospital, Nagoya, Japan., nagoya city, Aichi, Japan

## Abstract

**Background:**

*Enterobacter cloacae* complex (ECC) possesses intrinsic chromosomal AmpC β-lactamase, often complicating the detection of extended-spectrum β-lactamase (ESBL) production. Although ECC has received less attention than other Enterobacterales in the context of ESBL production, the increasing prevalence of highly resistant ECC strains warrants detailed molecular analysis.

**Figure:**
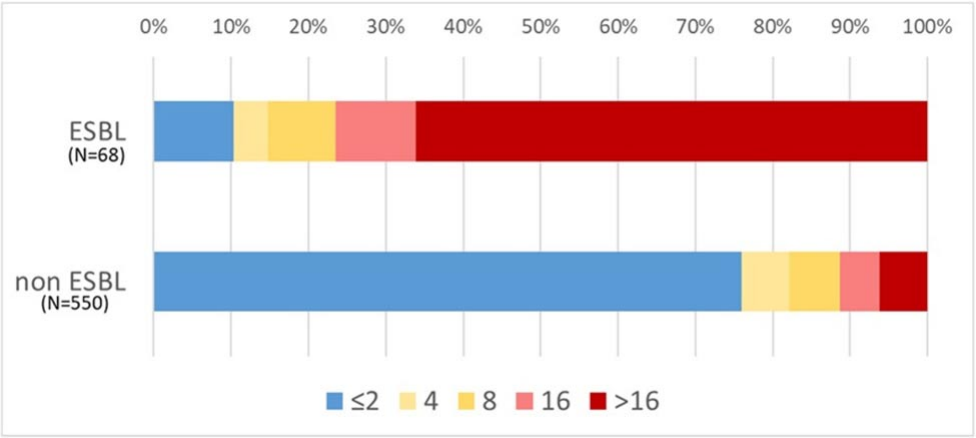
CFPM-MIC of ESBL-producing and non ESBL-prpducing highly resistant Enterobacter cloacae complex (ECC) isolates Distribution of CFPM-MIC is higher in ESBL-producing ECC isolates than non ESBL-producing ECC

**Methods:**

This single-center, retrospective study analyzed ECC isolates detected between 2015 and 2021. Among 1,757 ECC strains subjected to susceptibility testing, 618 highly resistant isolates—defined by resistance to third-generation cephalosporins (CTX, CTRX, or CAZ)—were analyzed for β-lactamase phenotypes using inhibitor-based disk potentiation method. Sixty-eight isolates suspected of ESBL production underwent whole-genome sequencing (WGS). Chi-square tests were used for statistical analysis.

**Results:**

Highly resistant ECC accounted for 35.2% of isolates (618/1,757). Among these, phenotypic classification revealed AmpC-type (86%), ESBL-type (9%), MBL-type (2%), and AmpC+ESBL (2%). ESBL-producing strains exhibited significantly higher resistance to piperacillin/tazobactam, cefepime, fluoroquinolones, aminoglycosides, and trimethoprim-sulfamethoxazole (p < 0.01). WGS of 68 ESBL-suspected strains identified *E. hormaechei* subsp*.xiangfangensis*/*steigerwaltii*(53%) as the most common subspecies, with CTX-M-15 being the predominant ESBL genotype. Clonal dissemination of ST133 and ST90 was observed.

**Conclusion:**

A considerable proportion of ECC isolates at our institution were ESBL producers, with broader resistance profiles beyond β-lactams. The presence of clonally related ESBL-producing strains highlights the need for continuous antimicrobial susceptibility and genomic surveillance and careful antimicrobial stewardship in managing ECC infections.

**Disclosures:**

All Authors: No reported disclosures

